# ZNF-Mediated Resistance to Imatinib Mesylate in Gastrointestinal Stromal Tumor

**DOI:** 10.1371/journal.pone.0054477

**Published:** 2013-01-25

**Authors:** Lori Rink, Michael F. Ochs, Yan Zhou, Margaret von Mehren, Andrew K. Godwin

**Affiliations:** 1 Department of Medical Oncology, Fox Chase Cancer Center, Philadelphia, Pennsylvania, United States of America; 2 Departments of Oncology and Health Science Informatics, School of Medicine, Johns Hopkins University, Baltimore, Maryland, United States of America; 3 Department of Statistics, Fox Chase Cancer Center, Philadelphia, Pennsylvania, United States of America; 4 Department of Pathology and Laboratory Medicine, University of Kansas Medical Center, Kansas City, Kansas, United States of America; 5 The University of Kansas Cancer Center, University of Kansas Medical Center, Kansas City, Kansas, United States of America; University of Pittsburgh Cancer Institute, United States of America

## Abstract

Although imatinib mesylate (IM) has transformed the treatment of gastrointestinal stromal tumors (GIST), many patients experience primary/secondary drug resistance. In a previous study, we identified a gene signature, consisting mainly of Kruppel-associated box (KRAB) domain containing zinc finger (ZNF) transcriptional repressors that predict short-term response to IM. To determine if these genes have functional significance, a siRNA library targeting these genes was constructed and applied to GIST cells *in vitro*. These screens identified seventeen “IM sensitizing genes” in GIST cells (sensitization index (SI) <0.85 ratio of drug/vehicle) with a false discovery rate (FDR) <15%, including twelve ZNF genes, the majority of which are located within the HSA19p12–13.1 locus. These genes were shown to be highly specific to IM and another tyrosine kinase inhibitor (TKI), sunitinib, in GIST cells. In order to determine mechanistically how these ZNFs might be modulating response to IM, RNAi approaches were used to individually silence genes within the predictive signature in GIST cells and expression profiling was performed. Knockdown of the 14 IM-sensitizing genes (10 ZNFs) universally led to downregulation of six genes, including TGFb3, periostin, and NEDD9. These studies implicate a role of KRAB-ZNFs in modulating response to TKIs in GIST.

## Introduction

Gastrointestinal stromal tumors (GISTs) are the most common mesenchymal tumors with an estimated annual incidence of ∼6,000 new cases in the United States [1]. Mazur and Clark originally described these tumors in 1983, noting they contained smooth muscle and neural elements [2]. GISTs are recognized immunohistochemically by CD117, the 145 kDa transmembrane glycoprotein KIT. They are believed to arise from the Interstitial Cells of Cajal (ICC) [3] or from interstitial mesenchymal precursor stem cells [4]. The majority (∼70%) of GISTs possess gain-of-function *KIT* mutations in exons 9, 11, 13 or 17. Smaller subsets of GISTs possess either gain-of-function mutations in *PDGFRA* (exons 12, 14, or 18) (∼10%), *BRAF* (exon 15) (∼1–2%) or no mutations, commonly referred to as wild-type (WT) GISTs (∼10–15%) [5], [6] [1], [7]. Recently, germline inactivating mutations in the genes encoding succinate dehydrogenase subunits A, B, C and D (*SDHA*, *SDHB*, *SDHC* or *SDHD*) have been reported in GISTs lacking *KIT*/*PDGFRA* mutations [8], [9] correlating with overexpression of IGF1R in these tumors [10], [11], [12]. The most common primary site for these neoplasms is the stomach (∼55%) [1], [13], followed by small intestine (∼30%), colon, rectum, anus, esophagus, mesentery and omentum (∼15% total) [14], [15], [16]. GISTs occur most frequently in patients over 50, with a median age of presentation of 58 years; however, GISTs have also been observed in the pediatric population [15].

Imatinib (IM), an oral 2-phenylaminopyrimidine derivative that works as a selective inhibitor against mutant forms of type III tyrosine kinases such as KIT, PDGFRA, and BCR/ABL, has significantly improved the clinical outcome of patients with advanced GIST. IM has become the standard treatment for patients with metastatic and/or unresectable GIST, with objective responses or stable disease obtained in >80% of patients and a median time to progression of 2 years [17], [18], [19]. Response to IM has been correlated to the genotype of a given tumor [20]. GIST patients with exon 11 *KIT* mutations have the best response and disease-free survival, while other *KIT* mutation types and WT GIST have low response rates to IM. For patients whose GISTs fail IM therapy, sunitinib malate, a multi-targeted tyrosine kinase inhibitor, with activity against KIT, PDGFRA, VEGFR, and FLT-1, is utilized as second line therapy. Clinical trials of sunitinib have demonstrated an objective response rate of ∼10% with further disease stabilization in 50% of patients with IM-refractory disease, and a median progression-free survival of 6 months [21]. While it is not clear what the final result will be for agents currently in clinical trials of GIST patients, e.g., dasatinib, nilotinib, masatinib, and regorafenib, they too are likely to have limited action as monoagents.

Although IM has revolutionized the treatment of patients with GISTs; clinical resistance to IM has become a reality, despite the initial efficacy observed. Furthermore, very limited options exist for patients (∼20%) that are refractory at the start of treatment. We have focused on determining why some GISTs respond initially to IM, while others are refractory, sometimes regardless of mutational status. Using clinical pre-treatment biopsy samples from a prospective neoadjuvant phase II trial (RTOG 0132), we previously identified a 38-gene signature that includes KRAB-*ZNF 91* subfamily members that can predict rapid response to IM [22]. This was the first neoadjuvant trial of IM in GIST patients and the first molecular study to examine gene expression changes associated with tumor response following drug treatment in both primary and metastatic GISTs. Here we demonstrate that 17 of these IM-sensitizing genes, including 12 ZNFs, are not only predictive of IM response but mediate the drug's activity. In order to determine mechanistically how these ZNFs might be modulating response to IM, RNAi approaches were used to silence the expression of the genes within the predictive signature (including 10 ZNFs) in GIST cells and individually assess their effect on global gene expression in order to find common regulatory pathways.

## Methods

### Cell Cultures

GIST-T1, a tumor cell line possessing a heterozygous mutation in *KIT* exon 11 kindly provided by Takahiro Taguchi [7], [23], [24], and A2780 [25], an ovarian cancer cell line were grown as previously described [26]. For drug treatment, drugs were added directly to the cell medium at the indicated final concentration for the specified period of time. Imatinib mesylate (Gleevec™), ifosfamide, doxorubicin and sunitinib malate were purchased from the Fox Chase Cancer Center pharmacy.

### siRNA transfection and IM sensitivity

The custom siRNA library targeting 53 genes, 25 of which were identified in our previous study [22], was designed and synthesized with four independent siRNAs pooled for each target (Qiagen, Valencia, CA). Transfection conditions were determined for GIST T1 cells using siRNA smart pools against KIT and GL-2 (Dharmacon) controls to achieve Z′ factor of 0.5 or greater. We used a reverse transfection protocol in which the final concentration of siRNA was 50 nM. Forty-eight hours after transfection, vehicle only or vehicle+IM (45 nM) were added to two plates. After twenty-four hours cell viability was assessed using the Alamar blue assay. This assay is based on the ability of living cells to convert the redox dye, resazurin, into the fluorescent end product, resorufin. Alamar blue was added to all wells and incubated for three hours followed by data recording using an EnVision microplate reader (PerkinElmer). Hits identified by pooled siRNAs were validated by confirming that 2 or more (of 4) independent siRNAs (at 12.5 nM) targeting the same gene in each case provided sensitization to IM. Ifosfamide (1 µM), doxorubicin (100 nM) or sunitinib (40 nM) were also applied to the deconvoluted siRNAs to identify sensitizing targets.

### Statistical Analysis

Alamar blue raw luminescence values collected from the high throughput siRNAs screen were normalized to negative GL2 control samples on the same plate. Each siRNA was then assigned a sensitization index (SI) as SI = (V_drug_/GL2_drug_)/(V_DMSO_/GL2_DMSO_), where V was viability measure in wells transfected with targeting siRNA and GL2 was the median viability of five wells with non-targeting negative control siRNA on the same plate. All calculations were automated using the cellHTS2 [27] package within the Bioconductor open source software package (http://bioconductor.org) [28]. Drug effect was measured using a linear model [29] encoded in the limma R/Bioconductor package [30]. Hits were identified on the basis of statistical significance as well as biological significance. Statistical significance was determined from the linear model with Benjamini-Hochberg correction [31]. Hits showing an FDR of less than 15% were considered statistically significant. Biological significance was defined as a decrease in SI greater than 15%. Hits identified as statistically and biologically significant were further validated.

### RNA isolation & Microarray Gene Expression Arrays

Total RNA was isolated from cells using TRIzol reagent according to the protocols provided by the manufacturer (Invitrogen Corp., Carlsbad, CA). RNA quantification and quality assessment were assessed using the 2100 Bioanalyser (Agilent Technologies, Santa Clara, CA). Affymetrix Human Exon 1.0 ST arrays were used for hybridization, arrays were scanned using an Affymetrix GeneChip Scanner 3000 and the scanned images were quantified using Affymetrix GeneChip Command Console Software (AGCC). The expression data was obtained in the form of CEL files.

### Microarray Data analysis

CEL files were loaded into R/Bioconductor [28] and analyzed with the oligo package [32]. Data were normalized using RMA and summarized to the gene level using the core probes. The data comprised estimates for 22011 genes across 16 samples. Given the low number of control samples, we identified genes based on mean expression changes relative to controls. We defined a log2 fold change thresholds of 1.0. Then we focused on genes found to be down- or up-regulated in all samples with this cutoff, which included six downregulated genes all with mean log2 fold change less than −1.5. One gene was upregulated in all knockdowns beyond the threshold, and it had a mean log fold change of 1.3.

To explore the impact of the global changes in gene expression, we analyzed the data using a t-test comparing controls to siRNA knockdowns with pooled variance to account for the low number of control samples. Gene set analysis using a Wilcoxon gene set test from the limma R package [30] was used with the t-statistic to identify chromosomal regions and curated pathways (KEGG, Biocarta, Reactome) showing significant changes between knockdowns and controls. All significance was defined as Bonferroni corrected p-value<0.05. Microarray data are available through the Gene Expression Omnibus (accession number GSE40080).

### Quantitative RT-PCR

RNA was reverse transcribed to cDNA by SuperScript II reverse transcriptase (Invitrogen). Expression of mRNA for the 14 IM-sensitizing genes, periostin, TGFb3 and two endogenous control genes (HPRT and 18S) was measured in each sample by real-time PCR (with TaqMan Gene Expression Assay products on an ABI PRISM 7900 HT Sequence Detection System, Applied Biosystems, Foster, CA) following protocols recommended by the manufacturer and as previously described [33]. The relative mRNA expressions were adjusted with either HPRT or actin. The primer/probe (FAM) sets were obtained from Applied Biosystems.

## Results

### Functional Evaluation of a Predictive Gene Set Associated with Response to IM in GIST

**Figure 1:**
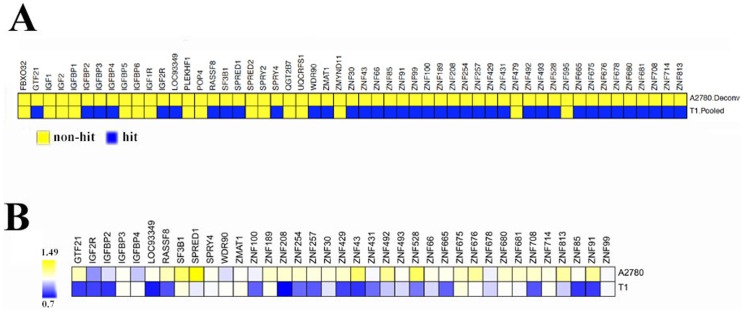
siRNA screen identifies GIST-specific IM sensitizing genes. (A) Primary IM sensitizing hits (SI<0.85, blue) identified using pooled siRNAs in A2780 (0 hits) and T1 cells (37 hits). (B) Validation of the primary IM sensitizing hits using deconvoluted siRNA screen. 17 of the primary hits were validated. Heatmap shows the average SI values from two or more confirmed individual siRNAs. SI values on log scale are plotted.

**Figure 2:**
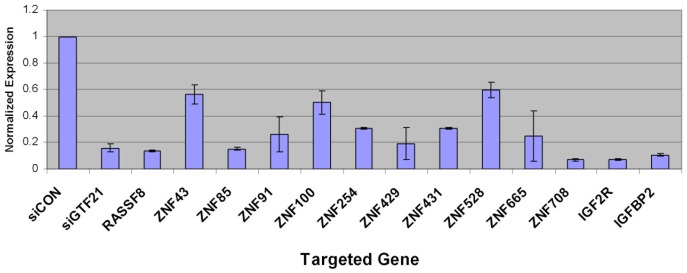
Validation of target gene knockdown by quantitative RT-PCR. RNA expression of indicated target genes in GIST T1 cells after knockdown with corresponding siRNA. Expression levels were normalized to HPRT. Results are of three independent experiments.

Using clinical samples from a prospective neoadjuvant/adjuvant phase II trial we had previously identified a genetic signature which includes KRAB-*ZNF 91* subfamily members that may be predictive of rapid response to short-term IM treatment and which may provide a prognostic biomarker identifying patients who will benefit from it [22]. This gene signature is composed of thirty-eight genes that were expressed at significantly lower levels in the pre-treatment samples of tumors that rapidly responded to IM. Eighteen of these genes encoded KRAB domain containing zinc finger (KRAB-ZNF) transcriptional repressors, ten of which of which mapped to a single locus on chromosome 19p. In order to determine if modifying expression of genes within this predictive signature could enhance the sensitivity to IM, we designed a custom siRNA library containing 53 genes, 25 of which showed differential expression between stable disease and rapid response groups in the aforementioned study (**Table S1**) [22]. There were thirteen genes identified in the previous study that were not represented in the siRNA library mainly because they were not annotated or had high homology to an already included ZNF. In addition to those 25 genes, we included 13 additional genes located in that same locus, i.e., HSA19p12–19p13.1 that showed a pattern of differential expression but did not reach statistical significance in our previous study (**Table S2**). The remaining 15 genes included in the current screen were of interest as a result of our previous studies that elucidated the role of IGF1R and KIT signaling in the pathogenesis of GIST, i.e., SPRY2 & 4, SPRED1 & 2, FBXO32, IGF1R, IGF2R, IGF1 & 2 and IGFBP1–6 (**Table S3**) [10], [11], [34]. We screened GIST T1 [23], an IM sensitive GIST cell line, and an unrelated ovarian cancer cell line, A2780 [25], with the custom siRNA library (four siRNAs pooled/gene) in combination with vehicle (PBS) or small molecule inhibitor, IM at IC_30_ concentration. Viability was measured with Alamar blue, a metabolic indicator of cell viability. From these screens, 37 of the 53 genes were identified as “primary IM sensitizing hits” in GIST T1 cells (**Figure 1A** **, bottom row, blue boxes**). These hits are defined as genes that when expression was silenced, decreased control-normalized viability by at least 15% in the presence of IM compared to the viability in the presence of vehicle (defined as the Sensitization Index (SI) <0.85) with a false discovery rate (FDR) <15%. No hits were identified in the A2780 cell line, indicating GIST-specificity (**Figure 1A** **, top row, yellow boxes**). Seventeen of the primary 37 hits were validated by confirming that 2 or more (of 4) independent siRNAs targeting the same gene in each case provided sensitization to IM (SI<0.85, FDR<15%) (**Figure 1B** **, bottom row**). Interestingly, 12 of the 17 (71%) validated hits were ZNF genes, 10 (59%) of which are located within the HSA19p12–13.1 locus. In addition, two members of the insulin-like growth factor (IGF) family, IGF2R and IGFBP2, were identified as sensitizing hits. Quantitative PCR analysis confirmed knockdown of the target in 15/17 validated genes (**Figure 2**).

### Drug Specificity of Sensitizing Genes

**Figure 3:**
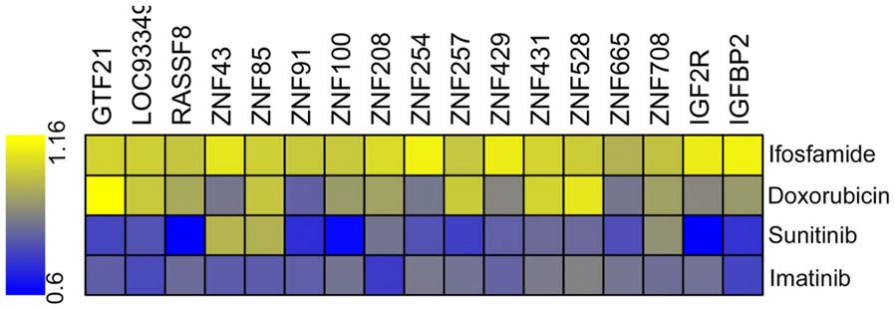
Heatmap showing sensitizing index (SI) ranging from 0.6 (blue) to 1.16 (yellow). Sensitization was tested in GIST T1 cells in the presence of four drugs including separately: Ifosfamide (top), Doxorubicin (second row), Sunitinib (third row) and IM (bottom). SI values on log scale are plotted.

The validation set has also been tested with other agents to measure IM specificity. We selected doxorubicin (anthracycline antibiotic, a DNA intercalating agent) and ifosfamide (nitrogen mustard alkylating agent), both relatively ineffective chemotherapeutic agents used prior to IM to treat GISTs, as well as sunitinib, a small molecule kinase inhibitor (targets KIT, PDGFRA, VEGFR, and FLT-1) which has shown some success in the treatment of GISTs. For this small screen, the best two siRNAs from the previous screen were pooled in a 96-well format, and cells were transfected and treated (at IC_30_ concentrations) as previously described. SI values were calculated as previously described. None of these genes were sensitizing hits for ifosfamide. Knockdown of 5 out of 17 (29%) of these genes sensitized GIST T1 cells to doxorubicin. In contrast, knockdown of 14 out of 17 genes (82%) sensitized to sunitinib (**Figure 3**). These findings are consistent with the fact that sunitinib has many of the same targets as IM and indicate that these sensitizing genes are highly specific to IM and sunitinib.

### Elucidating Targets of these “IM Sensitizing Genes”

**Figure 4:**
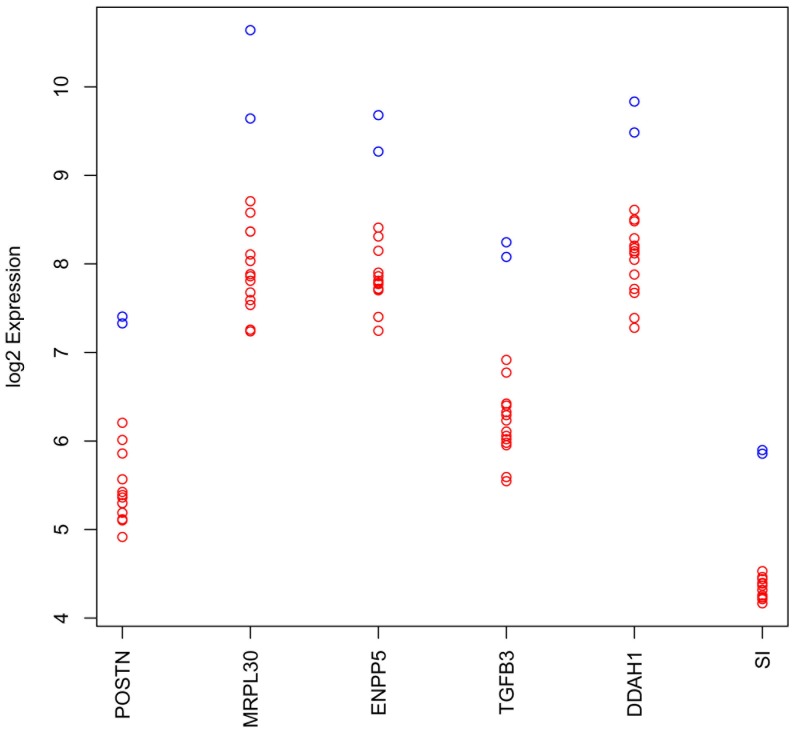
Log2 expression estimates for the six genes identified as downregulated by at least a factor of 2 in all samples relative to mean control value. For each gene, blue circles indicate log2 expression estimates for siCON samples and red circles indicate values for knockdown samples.

To identify downstream targets of these ZNFs in the hope of elucidating the mechanisms by which they are modulating response to IM, Affymetrix Exon 1.0 ST arrays were used to examine gene expression on GIST T1 cells that have been transfected with siRNAs against 14 of the “sensitizing genes” (10 of which were ZNFs) as well as siCON (non-targeting siRNA). All samples were compared to the GIST T1 cells transfected with siCON to identify genes that changed on knockdown, and we generated our candidate list using minimum log2 fold change of 1.0 (equivalent to a halving of expression). Six genes were found to be downregulated by this amount in all samples with median log2 fold changes >−1.5. Due to other ongoing studies, we also identified NEDD9, which was downregulated in all samples though not above our threshold with a mean log fold change of −0.91 (**Table 1** **,** **Figure 4**). These data have been confirmed by qRT-PCR (**data not shown**). In addition, only one gene, *TMCO1*, was upregulated (log fold change = +1.33) universally across the samples with these thresholds (**Table 1**). These data are intriguing since two of the downregulated genes, periostin and NEDD9, have been shown to be transactivated by a third downregulated gene, TGFb3 [35], [36].

**Table 1:** Differentially expressed genes in all 14 samples.

Gene Symbol	Description	Cytoband	Mean LogFold Change
POSTN	periostin, osteoblast specific factor	13q13.3	−2.21
MRPL30	mitochondrial ribosomal protein L30	2q11.2	−1.98
ENPP5	ectonucleotide pyrophosphatase/phosphodiesterase 5	6p12.3	−1.95
TGFb3	transforming growth factor, beta 3	14q24.3	−1.65
DDAH1	dimethylarginine dimethylaminohydrolase 1	1p22.3	−1.62
SI	sucrase-isomaltase	3q26.1	−1.53
NEDD9/HEF1/Cas-L	Human enhancer of filamentation	6p25-p24	−0.91
TMCO1	transmembrane and coiled-coil domains 1	1q24.2	+1.33

### Pathway Analysis

**Figure 5:**
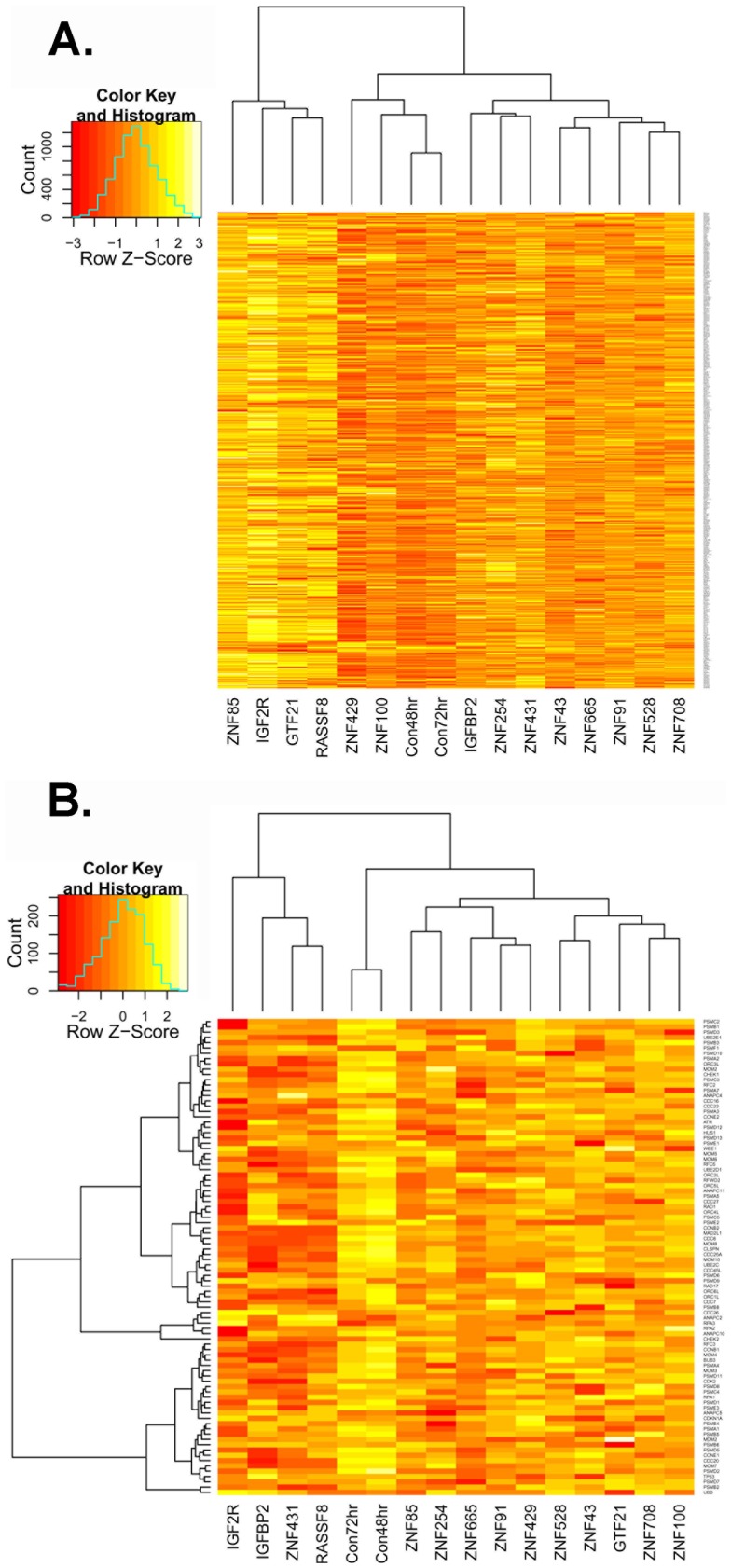
Heatmaps of relative expression levels for genes in two gene sets using row (gene) averaging to provide relative differences. (A) The heatmap for the genes at 19q13 are shown clustered by sample. The four samples on the left (knockdowns of ZNF85, IGF2R, GTF21, and RASSF8) show the strongest upregulation of gene relative to the siCON samples (Con48 hr, Con72 hr). Knockdowns of ZNF429 and ZNF100 behave much more like controls, as shown by the dendrogram, than do other knockdowns. (B) The heatmap for the Reactome Cell Cycle Checkpoints gene set is provided with clustering on both columns and genes. There is overall downregulation of cell cycle checkpoint genes in knockdowns relative to siCON samples, with knockdowns of IGF2R, IGFBP2, ZNF431, and RASSF8 showing the largest overall changes. In this case, no knockdown samples cluster closely with the controls.

Gene set analysis of t-statistics revealed significant changes at chromosomal locations 19p13, 16q13, 19q13, 3p21, 17q21, 11p15, and 6p22. As 19q13 was by far the strongest signal (corrected p<4.4×10^−21^), we examined changes in expression for all genes in the locus (**Figure 5A**). As can be seen, most siRNA knockdowns show slightly increased expression across the full locus relative to controls, with ZNF85, IGF2R, GTF21, and RASSF8 knockdowns showing the strongest signals, and ZNF429 and ZNF100 being most similar to controls.

A similar analysis of curated KEGG, Biocarta, and Reactome pathways identified nine pathways as significantly changed after Bonferroni multiple testing correction: KEGG Neuroactive Ligand Receptor Interaction, KEGG ECM Receptor Interaction, Reactome Cell Cycle Checkpoints, Reactome Cell Cycle Mitotic, Reactome M G1 Transition, Reactome Mitotic Prometaphase, Reactome Trasmembrane Transport of Small Molecules, Reactome Transmission Across Chemical Synapses, Reactome Mitotic M M G1 Phases. Most significant pathways involve cell cycle changes. We looked at all genes in the overarching class (Reactome Cell Cycle Checkpoints), which shows significant downregulation of checkpoint genes in all knockdowns relative to controls, with the strongest signals in knockdowns of IGF2R, IGFBP2, ZNF431, and RASSF8 (**Figure 5B**).

## Discussion

As a follow-up to our 2009 article [22] reporting the KRAB-ZNF-centric gene signature that predicts rapid response to IM in GIST, we set out to determine whether these genes could have a functional role in modulating drug response. We designed a customized siRNA library containing many members of the predictive gene signature as well as some additional genes that were of interest in other areas of focus. We found that seventeen of these predictive genes, when silenced, conferred sensitivity to TKIs, IM and sunitinib, in GIST cells. Gene set analysis revealed that the siRNA knockdowns affected many of the genes within the HSA19p12–19p13.1 locus previously identified as biomarkers of initial tumor response to imatinib. In order to more thoroughly determine the biological effects of the knockdowns, we performed gene set analysis on manually curated KEGG, Biocarta, and Reactome pathways. We determined that cell cycle pathways were highly affected and further analysis demonstrated that downregulation of cell cycle checkpoint genes appeared to play a role, suggesting a potential mode of action. These findings are intriguing and indicate potential therapeutic implications in GIST. Interestingly, Strauss et al. [37] recently showed significant correlations with these ZNFs and SUV and SUV_max_ as determined by dynamic PET studies. In addition, two recent reports have implicated ZNFs in modulation of therapeutic response in cancer cells. Duan et al. [38] showed overexpression of ZNF 93 and ZNF 43 in two human chondrosarcoma cell lines resistant to ET-743 (trabectedin; Yondelis) and PM00104 (Zalypsis), compared to their parental sensitive counterparts. They also found ZNF 93 overexpressed in two ET-743 resistant Ewing sarcoma cell lines and in a cisplatin-resistant ovarian cancer cell line. They went on to show that these cells could be sensitized by silencing ZNF 93. Although not TKIs, ET-473 and PM00104 both have shown activity in a variety of solid and hematological tumors. In addition, a 2012 report from Seyhan et al. [39], implicated a number of ZNFs in modulating response to neratinib, a TKI of the ERBB family, in SKBR-3, a breast cancer cell line overexpressing ERBB2. Also, of great interest is the fact that the majority (59%) of ZNFs identified from our siRNA screen are located within the 19p12–13.1 locus and belong to the ZNF 91 subfamily. 19p12–13.1 has recently been described as a breast and ovarian cancer susceptibility locus [40], [41], [42].

Not much is currently known regarding the function of the ZNF 91 subfamily members, which include 64 genes, 37 of which are found on chromosome 19 [43]. In an attempt to elucidate for the first time the down-stream targets of these ZNFs, RNAi approaches were used to silence these IM-sensitizing genes (including 10 ZNFs) in GIST-T1 cells followed by expression profiling with Affymetrix Human Exon 1.0 ST arrays. One interesting discovery arising from these studies is that the knockdown of the 14 IM-sensitizing genes universally led to downregulation of 3 genes, TGFb3, periostin, and NEDD9. Interestingly, TGFb3 is a known inducer of both periostin and NEDD9, both of which have been reported as frequently overexpressed in a variety of human cancers [35], [36], [44], [45], [46]. Periostin, also called osteoblast-specific factor 2, is a secreted protein originally isolated from osteoblast cells and has been shown to mediate cell adhesion as an extracellular matrix-associated protein [47] Periostin was subsequently shown to play a role in new bone formation and wound healing, normally regulated by TGFb [48]. There are a number of studies identifying periostin as overexpressed in a wide variety of tumor tissues including breast [45], pancreas [49], ovary [50], colon [51] and head and neck [52]. The PI3-K/AKT pathway has been described in several reports to be the critical pathway involved in regulation of periostin-induced tumorigenesis and prevention of stressed-induced tumor cell apoptosis in both colon and pancreatic cancer [49], [51]; specifically, periostin can induce the phosphorylation of AKT by binding a6b4 integrins [49]. Upregulation of periostin was reported to be stimulated by hypoxia-responsive growth factors, TGFa and bFGF, followed by activation of the PI3-K signaling pathway [35]. This is fascinating because hypoxia is a common condition within solid tumors, allowing cancer cells to undergo genetic and adaptive changes leading to escape from apoptosis and increased proliferation and survival [53], [54]. Also of interest, TGFb3 was recently described as a candidate gene for resistance of human glioblastoma multiforme cell lines to erlotinib, another tyrosine kinase inhibitor targeting EGFR [55]. NEDD9 (also known as HEF1, Cas-L) is a scaffolding protein whose overexpression has been shown to promote cell growth, migration and invasion in a number of cancers like melanoma and glioblastoma [46], [56], [57]. Intriguingly, like periostin, TGFb is known to induce transcription of NEDD9 mRNA [58], [59] and NEDD9 expression has been shown to be regulated by hypoxia [60], [61]. Therefore, it is plausible to imagine both periostin and NEDD9 regulated in a similar mechanism by TGFb and hypoxia, controlled by ZNFs, to modulate IM response in GIST cells. In addition, NEDD9 was shown to be overexpressed and SRC hyper-activated in an IM-resistant GIST cell line; when NEDD9 was knocked down, IM sensitivity was restored [62].

In addition to the ZNFs, IGF2R and IGFBP2, were identified as IM sensitizing genes in our study. Several reports, from our lab and others, have demonstrated roles for IGF family members in GIST pathogenesis and response to IM, prompting many groups to begin evaluating the use of IGF inhibitors as a potential therapy for GIST [10], [11], [63], [64]. The findings reported in this study lend further support for the testing of IGF inhibitors in GIST. Also of interest, IGF2R has been shown to directly complex with the latent form of TGFb contributing to its activation [65], [66]. Therefore, it is not surprising that knockdown of IGF2R leads to downregulation of TGFb3 and indicates that TGFb3, independent of the ZNFs, may be of importance in response to IM in GIST.

The studies reported here, for the first time, define the functional role of a family of KRAB-ZNF transcriptional repressors in IM sensitivity. These results also uncover that several members of the IGF signaling pathway, IGF2R and IGFBP2 are similarly associated with IM sensitivity in KIT mutant GIST cells. This is interesting given aberrant IGF signaling is a key oncogenic driver in 10 to 15% of GISTs that lack tyrosine kinase mutations and are less sensitive to IM and Sunitinib [10], [11]. This report describes potential downstream targets of a family of KRAB-ZNF, potent transcriptional repressors that for the most part remain completely uncharacterized. Overall, these studies implicate a role of KRAB-ZNFs in modulating response to TKIs and that TGFb3 may be a key downstream mediator of these proteins and thus IM sensitivity in GIST.

## Supporting Information

Table S1 Target genes in the custom siRNA library chosen by previous microarray study [22].
(DOC)

Table S2 Additional target genes from chromosome 19 showing differential expression but not reaching statistical significance in previous study [22].
(DOC)

Table S3 Target genes of additional interest [10], [11], [34].
(DOC)
