# Supplementary material for: ZNF-Mediated Resistance to Imatinib Mesylate in Gastrointestinal Stromal Tumor
Source: PLoS One. 2013 Jan 25;8(1):e54477. doi: 10.1371/journal.pone.0054477 (PMC3556080; doi:10.1371/journal.pone.0054477)
Supplement: Table S1 — Target genes in the custom siRNA library chosen by previous microarray study [22]. (DOC) [file pone.0054477.s001.doc]

Supplemental Table 1. Target genes in the custom siRNA library chosen by previous microarray study [22]

| **Gene Symbol** | **Description** | **Cytoband** |
| --- | --- | --- |
| ZNF678 | zinc finger protein 678 | 1q42.13 |
| ZMYND11 | zinc finger, MYND domain containing 11 | 10p15.3 |
| RASSF8 | Ras association (RalGDS/AF-6) domain family 8 | 12p12.1 |
| WDR90 | WD repeat domain 90 | 16p13.3 |
| ZNF66 | zinc finger protein 66 | 19p12 |
| ZNF85 | zinc finger protein 85 | 19p12 |
| ZNF431 | zinc finger protein 431 | 19p12 |
| ZNF429 | zinc finger protein 429 | 19p12 |
| ZNF43 | zinc finger protein 43 | 19p12 |
| ZNF208 | zinc finger protein 208 | 19p12 |
| ZNF676 | zinc finger protein 676 | 19p12 |
| ZNF99 | zinc finger protein 99 | 19p12 |
| ZNF91 | zinc finger protein 91 | 19p12 |
| ZNF528 | zinc finger protein 528 | 19q13.33 |
| ZNF665 | zinc finger protein 665 | 19q13.41 |
| ZNF813 | zinc finger protein 813 | 19q13.41 |
| SF3B1 | Splicing factor 3b, subunit 1, 155kDa | 2q33.1 |
| LOC93349 | hypothetical protein BC004921 | 2q37.1 |
| UGT2B7 | UDP glucuronosyltransferase 2 family, polypeptide B7 | 4q13.2 |
| ZNF595 | zinc finger protein 595 | 4p16.3 |
| ZNF479 | zinc finger protein 479 | 7p11.2 |
| ZNF680 | zinc finger protein 680 | 7q11.21 |
| GTF2I | general transcription factor II, i | 7q11.23 |
| ZNF189 | zinc finger protein 189 | 9q31.1 |
| ZMAT1 | zinc finger, matrin type 1 | Xq22.1 |
