# Supplementary material for: ZNF-Mediated Resistance to Imatinib Mesylate in Gastrointestinal Stromal Tumor
Source: PLoS One. 2013 Jan 25;8(1):e54477. doi: 10.1371/journal.pone.0054477 (PMC3556080; doi:10.1371/journal.pone.0054477)
Supplement: Table S2 — Additional target genes from chromosome 19 showing differential expression but not reaching statistical significance in previous study [22]. (DOC) [file pone.0054477.s002.doc]

Supplemental Table 1. Additional target genes from chromosome 19 showing differential expression but not reaching statistical significance in previous study [22].

| **Gene Symbol** | **Description** | **Cytoband** |
| --- | --- | --- |
| PLEKHF1 | pleckstrin homology domain containing, family F (with FYVE domain) member 1 | 19q12 |
| POP4 | POP4 processing of precursor 4 | 19q12 |
| UQCRFS1 | ubiquinol-cytochrome c reductase | 19q12 |
| ZNF30 | zinc finger protein 30 | 19q13.11 |
| ZNF257 | zinc finger protein 257 | 19q13 |
| ZNF100 | zinc finger protein 100 | 19p12 |
| ZNF254 | zinc finger protein 254 | 19p12 |
| ZNF492 | zinc finger protein 492 | 19p12 |
| ZNF493 | zinc finger protein 493 | 19p12 |
| ZNF675 | zinc finger protein 675 | 19p12 |
| ZNF681 | zinc finger protein 681 | 19p12 |
| ZNF708 | zinc finger protein 708 | 19p12 |
| ZNF714 | zinc finger protein 714 | 19p12 |
