# Supplementary material for: ZNF-Mediated Resistance to Imatinib Mesylate in Gastrointestinal Stromal Tumor
Source: PLoS One. 2013 Jan 25;8(1):e54477. doi: 10.1371/journal.pone.0054477 (PMC3556080; doi:10.1371/journal.pone.0054477)
Supplement: Table S3 — Target genes of additional interest [10], [11], [34]. (DOC) [file pone.0054477.s003.doc]

Supplemental Table 1. Target genes of additional interest [10, 11, 34].

| **Gene Symbol** | **Description** | **Cytoband** |
| --- | --- | --- |
| FBXO32 | F-box protein 32 | 8q24.13 |
| SPRED1 | sprouty-related, EVH1 domain containing 1 | 15q14 |
| SPRED2 | sprouty-related, EVH1 domain containing 2 | 2p14 |
| SPRY2 | sprouty homolog 2 | 13q31.1 |
| SPRY4 | sprouty homolog 4 | 5q31.3 |
| IGF1 | insulin-like growth factor 1 (somatomedin C) | 12q23.2 |
| IGF2 | insulin-like growth factor 2 (somatomedin A) | 11p15.5 |
| IGF1R | insulin-like growth factor 1 receptor | 15q26.3 |
| IGF2R | insulin-like growth factor 2 receptor | 6q26 |
| IGFBP1 | insulin-like growth factor binding protein 1 | 7p13-p12 |
| IGFBP2 | insulin-like growth factor binding protein 2 | 2q33-q34 |
| IGFBP3 | insulin-like growth factor binding protein 3 | 7p13-p12 |
| IGFBP4 | insulin-like growth factor binding protein 4 | 17q12-q21.1 |
| IGFBP5 | insulin-like growth factor binding protein 5 | 2q33-q36 |
| IGFBP6 | insulin-like growth factor binding protein 6 | 12q13 |
